# Synergistic inhibition of lung cancer cells by EGCG and NF-κB inhibitor BAY11-7082

**DOI:** 10.7150/jca.34285

**Published:** 2019-10-21

**Authors:** Lingyu Zhang, Jing Xie, Ruihuan Gan, Zhangwei Wu, Huatian Luo, Xingyong Chen, Youguang Lu, Lixian Wu, Dali Zheng

**Affiliations:** 1 School of Pharmacy, Fujian Medical University, 1 Xueyuan Road, University Town, Fuzhou, 350122, China.; 2 Department of Preventive Dentistry, School and Hospital of Stomatology, Fujian Medical University, 246 Middle Yangqiao Road, Fuzhou, 350001, China.; 3 Key Laboratory of Stomatology of Fujian Province, School and Hospital of Stomatology, Fujian Medical University, 88 Jiaotong Rd, Fuzhou, 350004, China.; 4 Shengli Clinical Collage, Fujian Medical University, 134 East Street, Fuzhou, 350001, China.

**Keywords:** EGCG, BAY11-7082, NF-κB, Apoptosis

## Abstract

Lung cancer has a poor 5-year survival rate and is the leading cause of cancer-related deaths worldwide. Thus, the development of more efficient therapeutic strategies is urgently needed. Many studies have shown that EGCG, a major polyphenol found in green tea, has potential anticancer effects. The present study aims to investigate the molecular mechanism of EGCG-mediated inhibition of proliferation in lung cancer cells and to explore the effects of combined treatment with EGCG and an NF-κB inhibitor, BAY11-7082, in A549 and H1299 cells both* in vitro* and* in vivo*. Our results showed that EGCG inhibits cell proliferation and migration and induces apoptosis in A549 and H1299 cells at relatively high concentrations (IC50=86.4 µM for A549 cells and 80.6 µM for H1299 cells). These effects are partially achieved via inhibition of the NF-κB signaling pathway. Combined treatment with EGCG and BAY11-7082, a potent NF-κB inhibitor, shows significant synergistic effects at relatively low concentrations. The inhibition rate reached approximately 50% in cells treated for 72 h with 20 µM EGCG and 5 µM (A549 cells) or 2.5 µM BAY11-7082 (H1299 cells). This synergistic anti-tumor effect was also observed in a xenograft model. These results indicated that EGCG inhibits lung cancer cell proliferation by suppressing NF-κB signaling. Coadministration of EGCG and BAY11-7082 has a synergistic effect both* in vitro* and *in vivo* and may serve as a novel therapeutic strategy for lung cancer.

## Introduction

Lung cancer has a high mortality rate and is a leading cause of cancer-related death worldwide 1. Approximately 85% of patients with lung cancer are diagnosed with non-small-cell lung cancer (NSCLC), one of the most predominant pathological types of lung cancer 2. Although considerable progress has been made in the treatment of NSCLC, the 5‐year overall survival rate of patients with NSCLC remains poor. Moreover, the recurrence rate of this disease is high 3. Many drugs have been designed to target specific signaling molecules, however these are affiliated with serious adverse effects, which limit their utility in the clinic 4. Thus, it is critical to consider other preventive and therapeutic measures for lung cancer.

Green tea (Camellia sinensis) is a popular beverage worldwide 5. Tea consumption may be linked to low incidences of various pathological conditions, including cardiovascular disease, diabetes, obesity, and cancer. Out of all the constituents of green tea, commonly referred to as catechins, (-)-epigallocatechin-3-gallate (EGCG) is the most abundant and biologically active component 6. EGCG has been shown to inhibit carcinogenesis induced by a wide variety of compounds in rodent cancer models 7. EGCG inhibits multiple signal transduction pathways in cancer cells 8. *In vitro*, EGCG has been shown to inhibit growth and stimulate apoptosis in a number of human cancer cell lines including leukemia, melanoma, breast, lung, and colon 9, 10. EGCG has also been shown to be effective in inducing apoptosis and inhibiting metastasis in a variety of cancer cell lines, including breast, prostate, liver and lung cancer cells, via induction of cell-cycle arrest or activation of the mitogen-activated protein kinase cascade 11. These effects have been extensively studied *in vitro* to try to elucidate the potential mechanism(s) of action of EGCG. Based on well-documented results, it is clear that EGCG is a promising chemopreventive and therapeutic agent for the treatment of lung cancer 2, 12, 13. Our present study aims to investigate the molecular mechanism underlying the inhibition of proliferation by EGCG in lung cancer cells.

The nuclear factor-KB (NF-κB) pathway has recently emerged as a promising therapeutic target for anticancer drugs 14, 15**.** NF-κB transcription factors are crucial regulators of mechanisms associated with tumorigenesis and can achieve multifaceted functions through regulation of NF-κB target genes 6, 16**.** NF-κB target genes are associated with numerous hallmarks of cancer, including proliferation (e.g., MYC, Cyclin D1), survival (e.g., BCL-2, Bcl-XL) inflammation (e.g., COX2, TNF-α) and metastasis (e.g., TWIST1, MMP2) 17. As NF-κB regulates a panel of key oncogenes and pro-survival genes, this pathway has also been implicated in tumor initiation, progression, and resistance to chemotherapy 18. Genome-level cancer studies have revealed that the NF-κB pathway is aberrantly activated in many types of human cancer. For example, mutations in the NF-κB pathway are potentially involved in lung cancer 19**.** EGCG has been demonstrated to effectively impair cancer cell growth by downregulating NF-κB pathway activity 4.

EGCG has been tested in numerous cancer cell lines and some clinical trials 20, 21, which have revealed three major issues limiting its utilization including high instability, low bioavailability and inappropriate systemic release 22. Human tumors often upregulate NF-κB signaling to acquire resistance to chemotherapy 23,24, NF-κB inhibitors may also serve as chemo-sensitizing agents in combination therapies. Combinations of natural compounds have been used successfully in the treatment of various types of cancer to increase therapeutic effects lower required dosages and to reduce drug resistance and/or side effects 25. Therefore, to evaluate the effect of EGCG in combination with other agents, we explored the combination of EGCG with BAY11-7082. This combination was selected because EGCG can inhibit NF-κB while BAY11-7082, a specific NF-κB inhibitor, was identified as an inhibitor of cytokine-induced IκB phosphorylation 26. BAY11-7082 has been shown to suppress NF-κB signaling both *in vitro* and *in vivo* 23,27. This compound, though not clinically approved, has been studied in mouse lymphoma models, which demonstrated that small molecule inhibition of the NF-κB pathway can cause tumor regression 27. Based on this rationale, we hypothesized that inhibition of NF-κB signaling via cotreatment with EGCG and BAY11-7082 may increase therapeutic efficacy in lung cancer.

In the present manuscript, we demonstrate for the first time that EGCG inhibits cell proliferation and migration and induces apoptosis in A549 and H1299 cells at relatively high concentrations, partially via inhibition of the NF-κB signaling pathway. Moreover, we show that EGCG and BAY11-7082 act synergistically to inhibit growth in lung cancer cells. This suggests that combinatorial NF-κB-targeting therapeutic regiments may be a novel strategy for the treatment of lung cancer.

## Materials and Methods

### Cell lines and culturing

A549 (WT p53, noninvasive) and H1299 (p53 null, invasive) cells were cultured in RPMI-1640 (Thermo Fisher Scientific Inc, Waltham, MA, USA) containing 10% (v/v) fetal bovine serum (Welgene, Inc, Daegue, South Korea) and 1% penicillin-streptomycin (Life Technology, USA) at 37°C in 5% CO_2_ humidified incubator 27.

### Reagents

MTT was obtained from Sigma-Aldrich, USA. The Annexin V-FITC kit was purchased from BD Pharmingen, USA. Transwell plates were from Corning Incorporated, USA. Antibodies against caspase-3 (9662s), -9 (9915s); Bcl-2 (2870s); GAPDH (ab181602) and TBP (ab818) were purchased from Abcam, Cambridge, UK; NF-κB (8242s) and phosphorylated NF-κB p65 (3033s) were purchased from Cell Signaling Technology, USA. Polymerase chain reaction (PCR) kits were purchased from Sigma-Aldrich Chemical Company, St. Louis, Missouri. NF-κB inhibitor (BAY11-7082) and EGCG were provided by MCE, USA. EGCG and BAY11-7082 (BAY) were resuspended in dimethyl sulfoxide (DMSO) as 10 mmol/L stock solutions.

### Cell viability assay

Cells (5×10^3^ /well) were seeded in 96-well plates (Labserve, USA) and incubated with different concentrations of EGCG (0-300 μM) or BAY11-7082 (0-5 μM) for 48 h and 72 h. Following incubation, 20 μL of MTT solution (5 mg/ml in PBS) was added to each well, and the mixtures were incubated for 4 h at 37**℃**. Then, the medium was discarded, and 150 μL of DMSO was added to dissolve the formazan crystals. The absorbance of each sample was read at 540 nm using a microplate reader (Thermo Multiskan GO, USA). A combination index (CI) was calculated from pooled data from 3 individual experiments. The combination indexes (CI) for two agents were calculated by CompuSyn synergism/antagonism analysis software (Version 1.0, ComboSyn, Inc., Paramus, NJ, USA). CI values of <1, =1 and >1 indicated synergism, additivity and antagonism in combined agent action, respectively 28.

### Colony formation assay

The effects of EGCG and BAY11-7082 treatment on A549 and H1299 cell viability was measured by colony formation assay. A549 and H1299 cells were seeded into 12-well plates at 600 cells/well. After a one-day incubation, control and experimental groups were incubated for 7 days and then stained with 0.1% crystal violet. Visible colonies (more than 50 cells) were counted under a light microscope. Survival rates were calculated relative to the number of colonies in the control group. All assays were repeated three times.

### Annexin V-FITC/PI Double Staining

Cells were seeded in 6-well plates (1×10^6^ cells/mL) and exposed to EGCG or BAY11-7082 for 48 h. The cells were then stained using an Annexin V-FITC/PI double-fluorescence apoptosis detection kit (Biouniquer Technology) according to the manufacturer's instructions. Samples were analyzed using a FACSCalibur flow cytometer within 1 h of staining.

### Western blot analysis and quantification

Lung cancer cells treated with EGCG and BAY11-7082 were lysed in lysis buffer. Protein samples (40 μg) were separated using Bis-Tris gels. Proteins were then transferred to polyvinylidene difluoride membranes (Millipore, USA). Membranes were blocked with 10% nonfat milk, washed with TBST and then incubated with primary antibodies overnight at 4**℃.** After washing with TBST the membrane was incubated with horseradish peroxidase-conjugated secondary antibodies for 1 h. Finally, visualization of protein bands was performed using an ECL substrate reagent kit (GE Healthcare) on a Gel Doc XR imaging system (Bio-RAD, USA). β-actin was used as the internal reference.The gray value of each protein was determined using Quantity One software, followed by statistical analysis.

### Real-time quantitative PCR (qPCR)

Total RNA was extracted from lung cancer cells used TRIzol reagent (Invitrogen, USA). mRNA expression levels were determined by real-time qPCR according to the instructions. GAPDH was set as an internal reference to determine the relative expression levels of genes of interest. The sequences of all primers used for qRT-PCR are shown in Table 1. Master Mix (Toyobo Co. Ltd. Japan) was used to detect and semiquantify the expression of target genes. The expression levels of target genes in the experimental group relative to those in the NC group were calculated by the 2^-△△Ct^ method. Analysis of each gene was performed at least 3 times. The sequences of all primers used for qRT-PCR analysis are listed in Table 1.

### Cell migration and invasion assays

In the wound healing assay, 1×10^5^ cells/well were seeded in 24-well plates. Lung cancer cells were starved for 24 h in FBS-free medium and then scraped with a 10 µL pipette tip. Then, the medium was replaced with fresh medium containing EGCG and BAY11-7082. Cells were incubated for 24 h, and then each well was photographed under a microscope (Carl Zeiss, Germany). The open wound area was measured and percentages were calculated using TScratch software.

For the Transwell migration assay, 8×10^4^ cells/well were seeded into Transwell chambers with 2% BSA and 200 μL medium containing various concentrations of EGCG. Then, 500 μL complete RPMI1640 medium (with 20% v/v FBS), which served as a chemoattractant, was added to the lower well. After a 24 h incubation at 37**℃**, cells were fixed with methanol and stained with 0.1% crystal violet. Stained filters were photographed under a microscope (Carl Zeiss, Germany). Migrated cells were quantified by manual counting and are represented as a percentage of the control value.

### Luciferase Reporter Assay

Luciferase assays were performed according to the manufacturer's instructions. Briefly, the NF-κB promoter was subcloned into a pGL3-Basic luciferase expression vector (Genepharma). A549 and H1299 cells were seeded in 24-well plates and allowed to grow overnight. When the cell density reached approximately 80%, they were cotransfected with a firefly luciferase-expressing vector (NF-κB-Luc) and a Renilla luciferase-expressing vector (pRL-CMV vector, Promega). 6 h after transfection, cells were treated with 20 μM EGCG and 0.625, 1.25, or 2.5 μM BAY11-7082. After 48 h incubation, cell lysates were harvested and luciferase activity was measured using a dual-luciferase assay (Promega). Firefly luciferase activity in each group was normalized to Renilla luciferase activity.

### Immunofluorescence

Lung cancer cells were treated with drugs for 24 h in the logarithmic growth phase. Then, they were fixed, ruptured, blocked, and incubated with primary antibody followed by secondary antibody. Finally, the cells were placed on the High Content Imaging System (Thermo Fisher, USA) to detect P-NF-κB foci using an anti P-NF-κB mouse monoclonal antibody. Nuclei were counterstained with Hoechst 33342 (BestBio #BB-4135-1, Shanghai, China).

### Cellular fractionation

Extraction Reagentslasmic and Nuclear Protein Extraction Reagents were used according to the manufacturer's protocol (thermo#78833). All fractions were further centrifuged at 16000g for 20min at 4**℃.** The supernatant was collected.

### *In vivo* growth inhibition assay

All animal experiments complied with the ARRIVE guidelines and were carried out in accordance with the U.K. Animals (Scientific Procedures) Act, 1986 and associated guidelines, EU Directive 2010/63/EU for animal experiments.

To measure the *in vivo* activity of EGCG and BAY11-7082, 24 5-week-old female Balb/c athymic nude mice were purchased from the Experimental Animal Center of Shanghai Shrike. Lung cancer cells were collected in the logarithmic growth phase, and then 4×10^6^ cells were inoculated into the left forelimb of each nude mouse. Once tumor volumes reached ≥100 mm^3^, the mice were randomly divided into four different treatment groups (6 mice per group): a CTL group (PBS + 5% DMSO), an EGCG-only group (20 mg/kg in saline, injected i.p.), a BAY11-7082-only group (10 mg/kg in 5% DMSO +PBS, injected i.p.), and an EGCG plus BAY11-7082 group. Tumor volumes were calculated from two-dimensional Vernier caliper measurements using the equation a^2^×b/2, where a is the smallest measurement and b is the largest measurement. Tumor sizes and body weights were measured daily. Tumor volume data are presented as the mean ± SEM.

### IHC staining and evaluation

Xenograft tissues were sectioned at 4 μm thick. After being baked at 60°C for 2 h, the tissue sections were deparaffinized with dimethylbenzene and rehydrated in different concentrations of alcohol. Then the slides were heated at 95°C in 0.01M citrate buffer (pH=6.0), and 3% hydrogen peroxide was used to quench peroxidase activity for 20min. Next the sections were treated with normal goat serum, followed by incubation overnight with anti-Ki67 antibody (1:800 dilution; Abcam, Cambridge, MA, USA), anti-phospho NF-κB at 4°C. After being rinsed with phosphate buffered saline (PBS), the sections were incubated with a secondary antibody for 1h and stained with 3, 3′-diaminobenzidine (DAB; Zhongshan biotech, Beijing, China). After hematoxylin counterstain was completed, all the sections were dehydrated and sealed. Two experienced pathologists independently evaluated the percentage of positive tumor cells and its staining intensity.

### Statistical analysis

Significant differences were determined by Student's t test (parametric) for *in vitro* studies and by analysis of variance (ANOVA) for *in vivo* studies. Differences were considered statistically significant at P < 0.05. All analyses were carried out using GraphPad Prism from GraphPad Software (San Diego, CA).

## Results

### EGCG inhibits A549 and H1299 lung cancer cell proliferation and induces apoptosis

**Figure 1:**
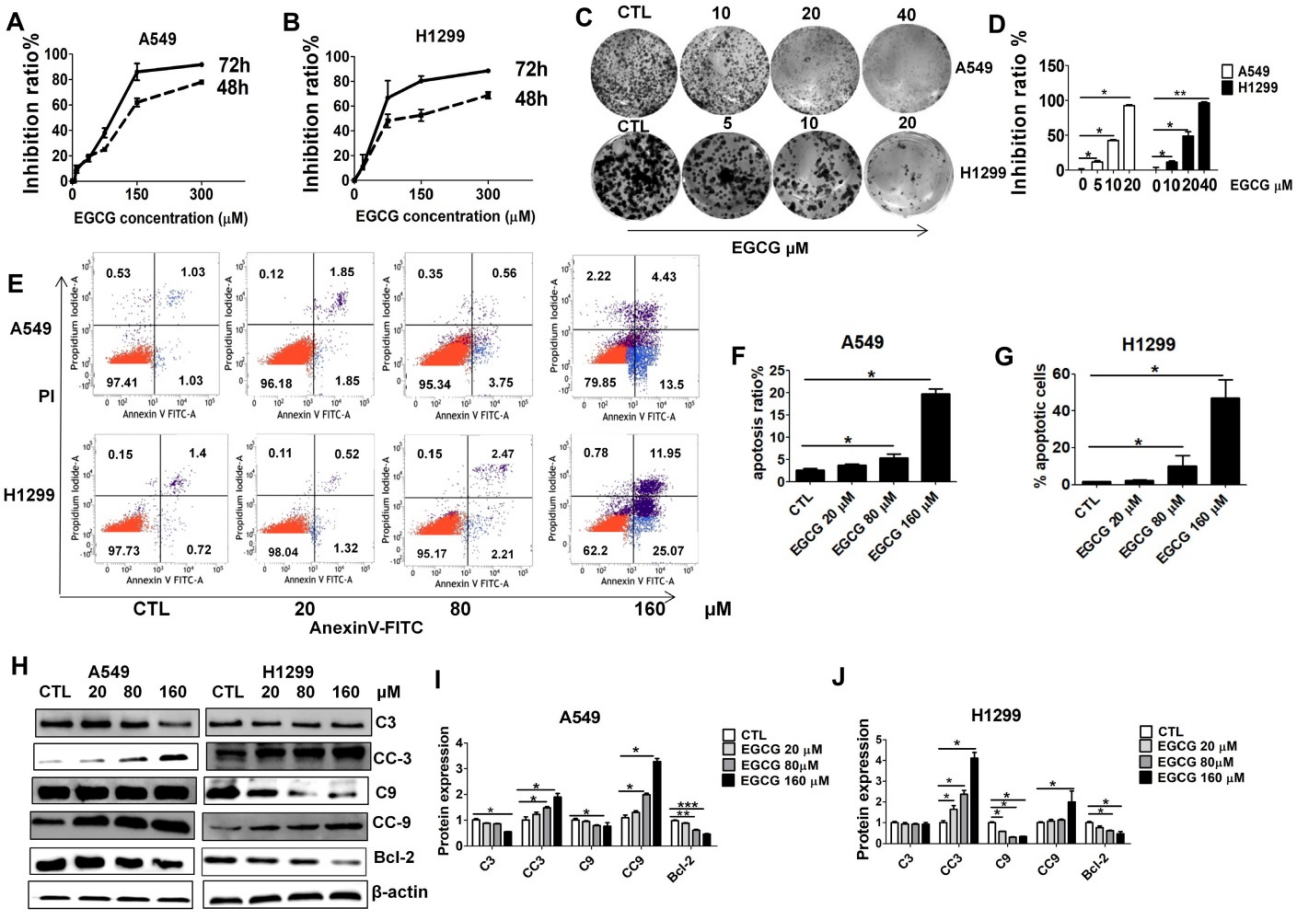
Growth inhibition and apoptosis induction in A549 and H1299 cells by EGCG. Growth inhibition curves A549 (A) and H1299 (B) cells exposed to various concentrations (20, 80 and 160 μM) of EGCG for 48 and 72 h, as determined using an MTT assay. Each value is expressed as the mean ± SD (n=3). (C) The effects of EGCG on colony formation in H1299 and A549 cells. (D) Quantification of colony formation assay. (E) Flow cytometry images. (F-G) Quantitative analysis of the percentage of apoptotic cells of EGCG after a 48 h incubation. The percentage of total apoptotic cells was defined as the sum of early and late apoptotic cells. (H-J) Western blot analysis of Caspase 3 (C3), cleaved Caspase 3 (CC3), Caspase 9 (C9), cleaved Caspase 9 (CC9), Bcl-2 and β-actin expression in A549 and H1299 exposed to different concentrations of EGCG.

Treatment with EGCG for 48 or 72 h resulted in the inhibition of cell proliferation in a time- and dose-dependent manner. As shown in Fig. 1, EGCG inhibited the growth of A549 cells at both 48 and 72 h, with an IC50 of 160.12 μM and 86.44 μM, respectively. H1299 cells were also sensitive to EGCG with IC50 values of 179.113 μM (48 h) and 80.566 μM (72 h). These results suggest that EGCG inhibits cell proliferation in both a concentration- and time-dependent manner (P <0.05). In addition, in the presence of 20 μM EGCG, lung cancer cells growth was weakly inhibited. Colony formation assays were then performed to validate the effects of EGCG on lung cancer cell growth. Colony forming efficiency was significantly reduced in the presence of 20 μM (H1299) and 40 μM (A549) EGCG (Fig. 1C-D).

To examine whether the reduced viability of lung cancer cells was caused by apoptosis, we carried out Annexin-V FITC/PI double staining and WB analysis. Annexin-V FITC/PI staining showed that when lung cancer cells were incubated with increasing doses of EGCG from 20 to 160 μM, the rates of cell apoptosis increased in a dose-dependent manner. The percentage of apoptotic A549 (H1299) cells upon 48 h treatment with 0, 20, 80 and 160 μM of EGCG was found to be 3.7% (2.5%), 4.5% (5.1%), and 22.2% (33.5%), respectively. EGCG concentrations less than 20 μM had only slight cytotoxic effects and caused minimal apoptosis (Fig. 1E-G). Furthermore, WB analysis showed that apoptosis significantly increased after 48 h of treatment with 160 µM (Fig. 1H-J) EGCG, as indicated by increased cleaved caspase-3 and -9 (CC3, CC9) and decreased Bcl-2.

### EGCG inhibits NF-κB signaling in A549 and H1299 cells

**Figure 2:**
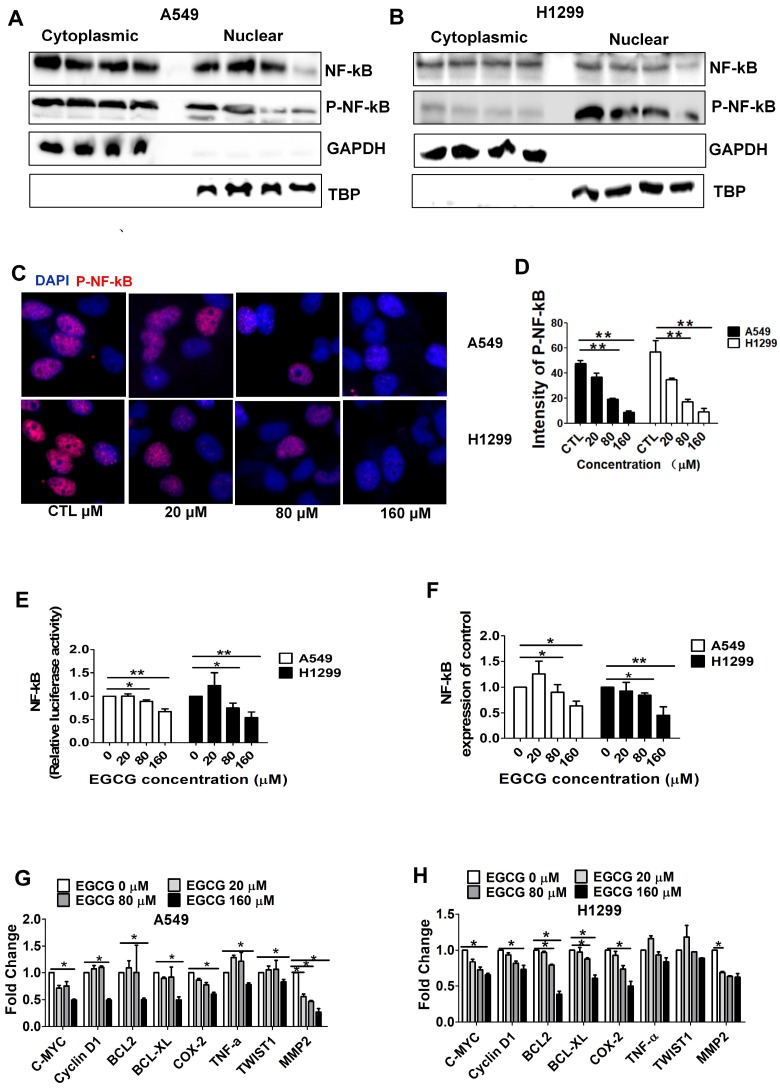
EGCG inhibits NF-κB in A549 and H1299 cells. (A-B) Western blot analysis of NF-κB, P-NF-κB in cytoplasmic and nuclear fractions of A549 and H1299 cells after treatment with EGCG. GAPDH (cytoplasmic) and TBP (nuclear) were used as controls. (C-D) P-NF-κB levels in cells were quantified by high-content imaging in three independent experiments. Representative photographs, ×20 for (C). Data are presented as the mean±SD of three independent experiments (D). (E) After transfection with NF-κB-Luc and Renilla plasmids, A549 and H1299 cells were lysed to measure firefly and Renilla luciferase activities in triplicate using a dual-luciferase assay kit (Promega). The firefly luciferase activity of each group was normalized to Renilla luciferase activity and is presented relative to that of the pGL-3 control group. Experiments were performed three times, and the mean ± SD of three experiments is shown. (F) qRT-PCR analysis of NF-κB mRNA expression in A549 and H1299 cells after treatment with indicated EGCG for 48 h. (G-H) qRT-PCR detection of NF-κB pathway-related gene expression in control cells and treated cells. The data are presented as the mean ± SE (n = 3) of fold-changes compared to the vehicle-treated A549 (G) and (H) H1299 cells. *P < 0.05; **P < 0.01.

EGCG may contribute to lung tumor regression by downregulating the expression of NF-κB, Bcl-xl, and Bax in lung cancer 29, 30. We thus hypothesized that EGCG inhibits NF-κB signaling in lung cancer cells. We performed nuclear protein extraction and examine NF-κB/P-NF-κB using Western blot assays to investigate the NF-κB activity affected by EGCG. As shown in Fig. 2A-B, WB analysis showed that treatment with 160 μM EGCG resulted in a significant decrease in P-NF-κB expression. Then we performed immunofluorescence assay of p-NF-κB to observe subcellular location. As expected, we did observe the p-NF-κB was significantly inhibited by 160 µM EGCG (Fig. 2C-D). Luciferase assays revealed that NF-κB activity was markedly reduced in lung cancer cells treated with 160 µM EGCG (Fig. 2E). Next, RT-PCR showed that the expression of NF-κB was significantly reduced by 160 µM EGCG (Fig. 2F). In addition, the expression of C-MYC, Cyclin D1, Bcl-2, Bcl-xL, COX-2, TWIST1, and MMP2, which are associated with NF-κB signaling, was analyzed to better understand the NF-κB pathways involved in inhibiting lung cancers and was obviously decreased by 160 μM EGCG (Fig. 2G-H). The data from this experiment revealed that downregulation of NF-κB expression is correlated with the anti-proliferative effects of EGCG in A549 and H1299 cells.

### Coadministration of EGCG and BAY11-7082 has a synergistic antiproliferative effect on A549 and H1299 cells

**Figure 3:**
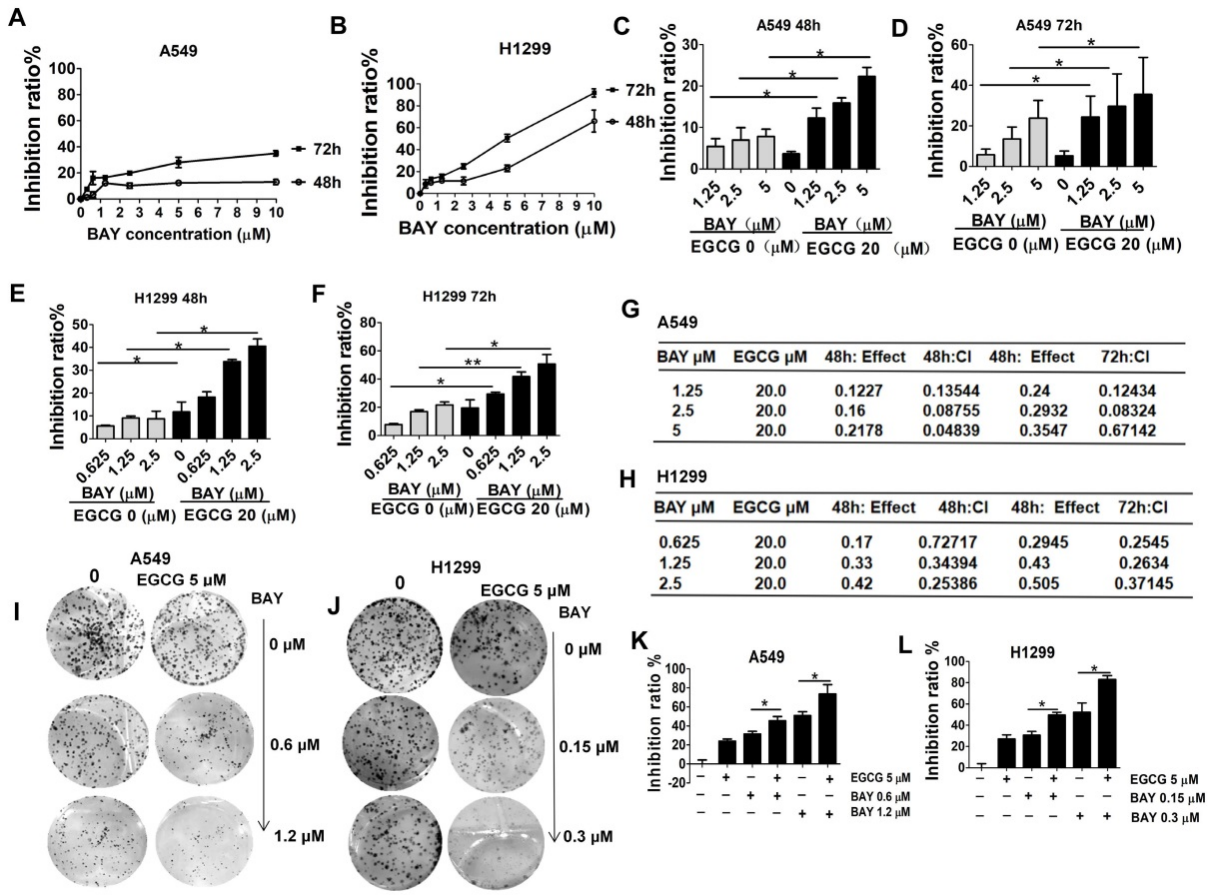
Cell growth is more effectively inhibited by cotreatment with EGCG and BAY11-7082 than by either agent alone. Growth inhibition curves A549 (A) and H1299 (B) cells exposed to various concentrations of BAY11-7082 for 48 and 72 h, as determined using an MTT assay. Each value is expressed as the mean ± SD (n=3). (C-D) A549 cells were treated with 20 µM EGCG; 1.25, 2.5, 5 µM BAY 11-7082; or the combination thereof for 48 and 72 h. Growth inhibition was significantly potentiated upon cotreatment with EGCG and BAY11-7082. (E-F) H1299 cells were treated with 20 µM EGCG; 0.625, 1.25, 2.5 µM BAY 117082; or the combination thereof for 48 or 72 h. (G-H) CI versus affected cell-fraction (fa) curves for both 48 and 72 h cotreatment with BAY-117082 and EGCG in A549 and H1299 cells. (I-L) The combined effects of EGCG and BAY11-7082 on colony formation in A549 and H1299 cells. Representative photographs of the clonogenic assay are presented in panels I-J. * P <0.05, **P< 0.01. The data are expressed as the mean ± S.D.

Since EGCG could inhibit lung cancer cell proliferation via downregulation of NF-κB as shown by MTT assays, CI index analyses and colony formation assays were performed to measure the proliferation of A549 and H1299 cells after treatment with EGCG and different concentrations of BAY11-7082 (a specific NF-κB inhibitor) to determine whether EGCG combined with BAY11-7082 has an synergistic effect. Besides, the cytotoxicity of BAY11-7082 on A549 and H1299 cells was also tested. The result showed BAY11-7082 at 5 µM only resulted in 12.8 % (48 h) and 21.3 % (72 h) inhibition in A549, and at 2.5 µM only resulted in 13.2 % (48 h) and 22.5% (72 h) inhibition in H1299 (Fig. 3A-B). Based on the above data, we used at a concentration of 20 µM EGCG (no cytotoxicity dose) and 0.625, 1.25, 2.5, 5 µM BAY11-7082 (lower concentration) in subsequent *in vitro* experiments. To investigate the effect of EGCG and BAY11-7082 on lung cancer cell growth, various doses and treatment lengths were investigated in A549 and H1299 cells (Fig. 3C-F). Cells were treated with EGCG and BAY11-7082 either alone or in combination for 48 and 72 h. The viability of lung cancer cells was significantly inhibited by cotreatment with BAY11-7082 and EGCG. To more accurately determine the effects (additive, synergistic, or antagonistic) of combination therapy, these results were evaluated mathematically using the method of Chou and Talalay 27. Subsequently, we found that EGCG and BAY11-7082 synergistically inhibited the viability of lung cancer cells, with a CI value of **<**1 in both A549 and H1299 cells (Fig. 3G-H). Furthermore, a colony growth assay confirmed that EGCG in combination with BAY11-7082 conferred marked repression of colony growth (Fig. 3I-L). These results were well consistent with the data obtained by the MTT assay in which cells were treated for 48 and 72 h as described above.

### Co-treatment with EGCG and BAY11-7082 induces apoptosis in lung cancer cells at a greater rate than treatment with either drug alone

**Figure 4:**
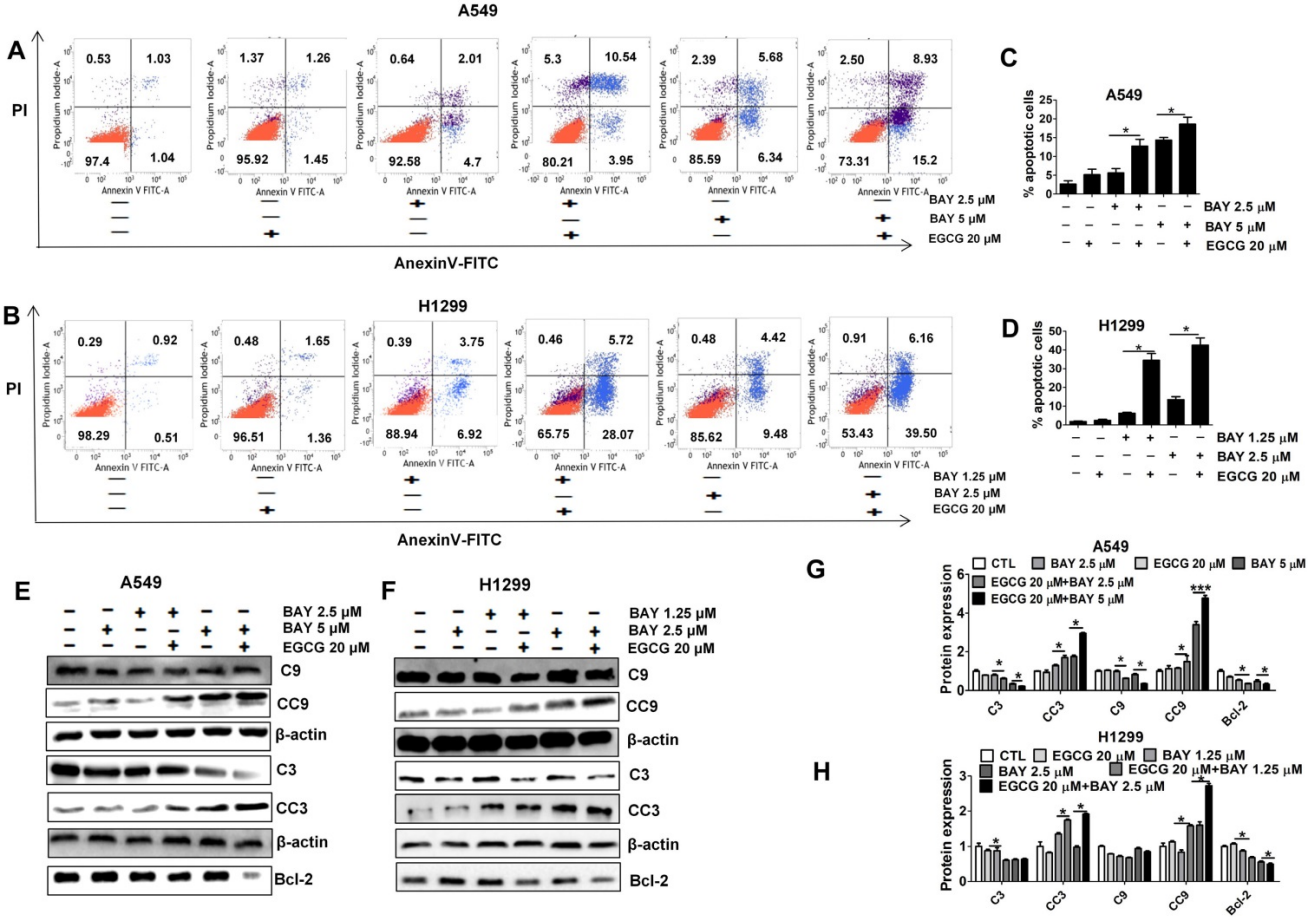
Combined treatment with EGCG and BAY11-7082 induces apoptosis in A549 and H1299 cells. (A-B) Flow cytometry images. (C-D) Quantitative analysis of the percentage of apoptotic cells after EGCG and BAY11-7082 treatment for 48 h. The percentage of total apoptotic cells was defined as the sum of both early and late apoptotic cells. (E-F) Representative Western blotting images of proteins collected from treated A549 and H1299 cells. (G-H) Statistical analysis of C3, CC3, C9, CC9, Bcl-2 and β-actin protein expression in A549 and H1299 cells after treatment. * P <0.05, ** P< 0.01, *** P < 0.001. Data are expressed as the mean ± S.D.

To test whether the increased response to BAY11-7082 and EGCG seen *in vitro* was associated with apoptosis, the apoptosis rates in each group were measured. We carried out annexin V-FITC/PI double staining. As shown in Fig. 4A-D, cells treated with both agents displayed much higher rates of apoptosis than cells treated by a single agent or control cells. Furthermore, Western blotting demonstrated that apoptosis was significantly increased after cotreatment for 48 h as indicated by increased CC3 and CC9. Bcl-2 was significantly reduced in the combination treatment group compared to the single-agent treatment groups and the control group (Fig. 4E-H). These results suggest that the decreased viability of lung cancer cells treated with BAY11-7082 and EGCG is partly due to the induction of apoptotic cell death and also imply a synergistic effect of EGCG and BAY11-7082.

### The expression and activity of NF-κB is decreased by dual inhibition by EGCG and BAY11-7082

**Figure 5:**
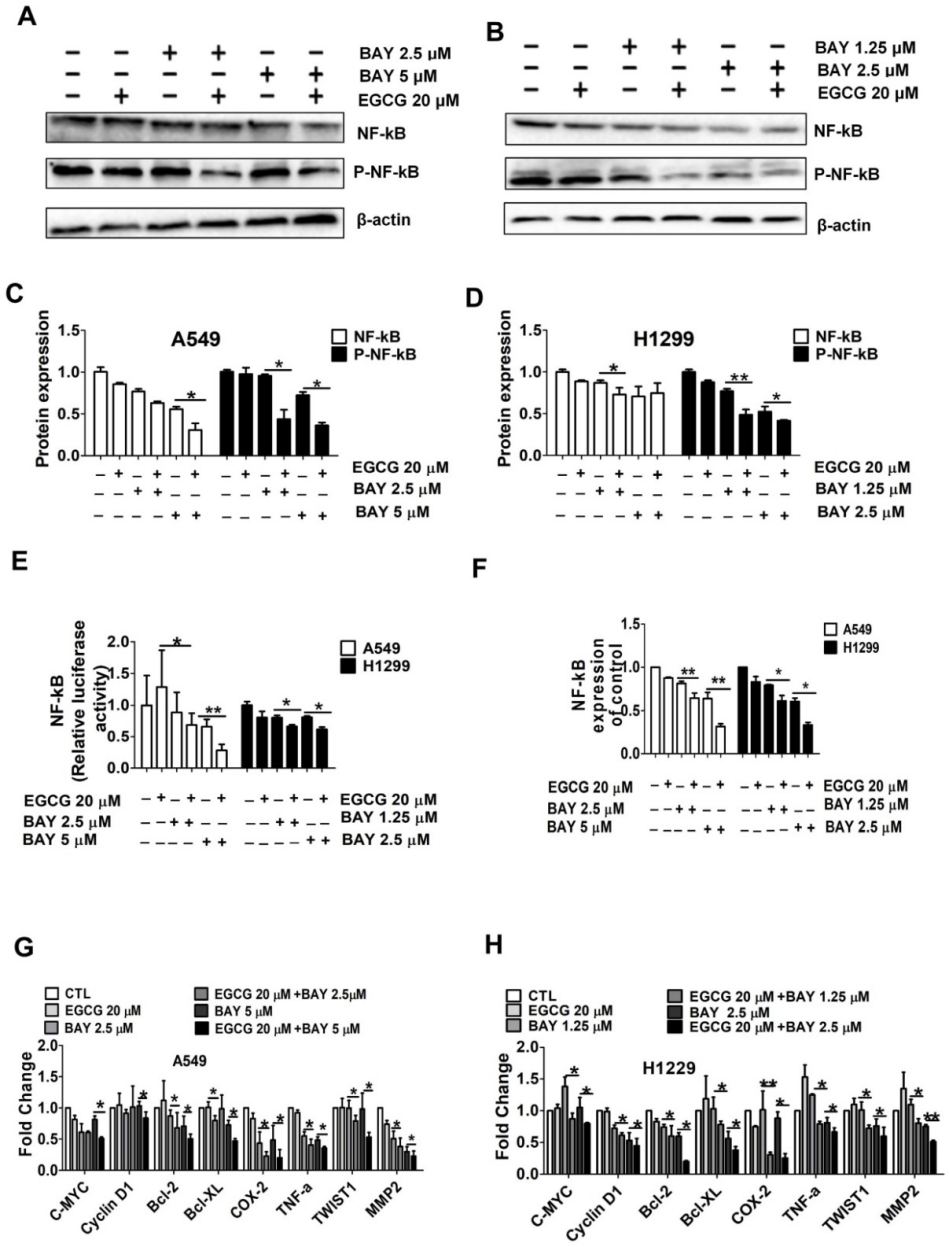
Cotreatment with EGCG and BAY11-7082 significantly inhibits NF-κB signaling. (A-D) Western blot analysis of total NF-κB and phosphorylated NF-κB p65 in A549 and H1299 whole cell lysates after treatment with 20 µM EGCG; 0.625, 1.25, or 2.5 μM BAY 11-7082; or the combination thereof for 48 h. (E) A549 and H1299 cells were cotransfected with SV40 and SV40 for dual luciferase reporter assays. (F) qRT-PCR analysis of NF-κB expression levels. (G-H) qRT-PCR analysis of C-MYC, Cyclin D1, Bcl-2, Bcl-xL, COX-2, TNF-α, TWIST1, and MMP2 mRNA expression in A549 and H1299 cells after treatment with 20 µM EGCG; 0.625, 1.25, or 2.5 μM BAY11-7082; or the combination thereof for 48 h. Data are shown as the mean ± S.D. (n=3). * P < 0.05, ** P < 0.01, ***P< 0.001.

To further explore the molecular mechanisms underlying the enhanced inhibition of cell viability by combination treatment, we performed Western blotting to detect the activation of NF-κB, a signaling protein that plays important roles in cell survival and apoptosis. As shown in Fig. 5A-D. NF-κB phosphorylation, an indicator of NF-κB activation 31, was not downregulated by EGCG (20 μM) and was only slightly downregulated by BAY11-7082. However, the combination treatment resulted in a significant downregulation of NF-κB phosphorylation. Moreover, in A549 and H1299 cells, combined treatment with EGCG and BAY11-7082 significantly induced NF-κB activity compared with BAY11-7082 treatment alone (Fig. 5E). In addition, we examined the expression of NF-κB pathway-related genes that are regulated by NF-κB inhibitors. RT-PCR analysis showed that the expression of NF-κB, C-MYC, CyclinD1, Bcl-2, Bcl-XL, COX-2, TNF-α, TWIST1 and MMP2 was significantly reduced by the combination treatment group compared to either single-agent treatment group (Fig. 5F-H).

### Cotreatment with EGCG and BAY11-7082 inhibits A549 and H1299 cell migration and invasion

**Figure 6:**
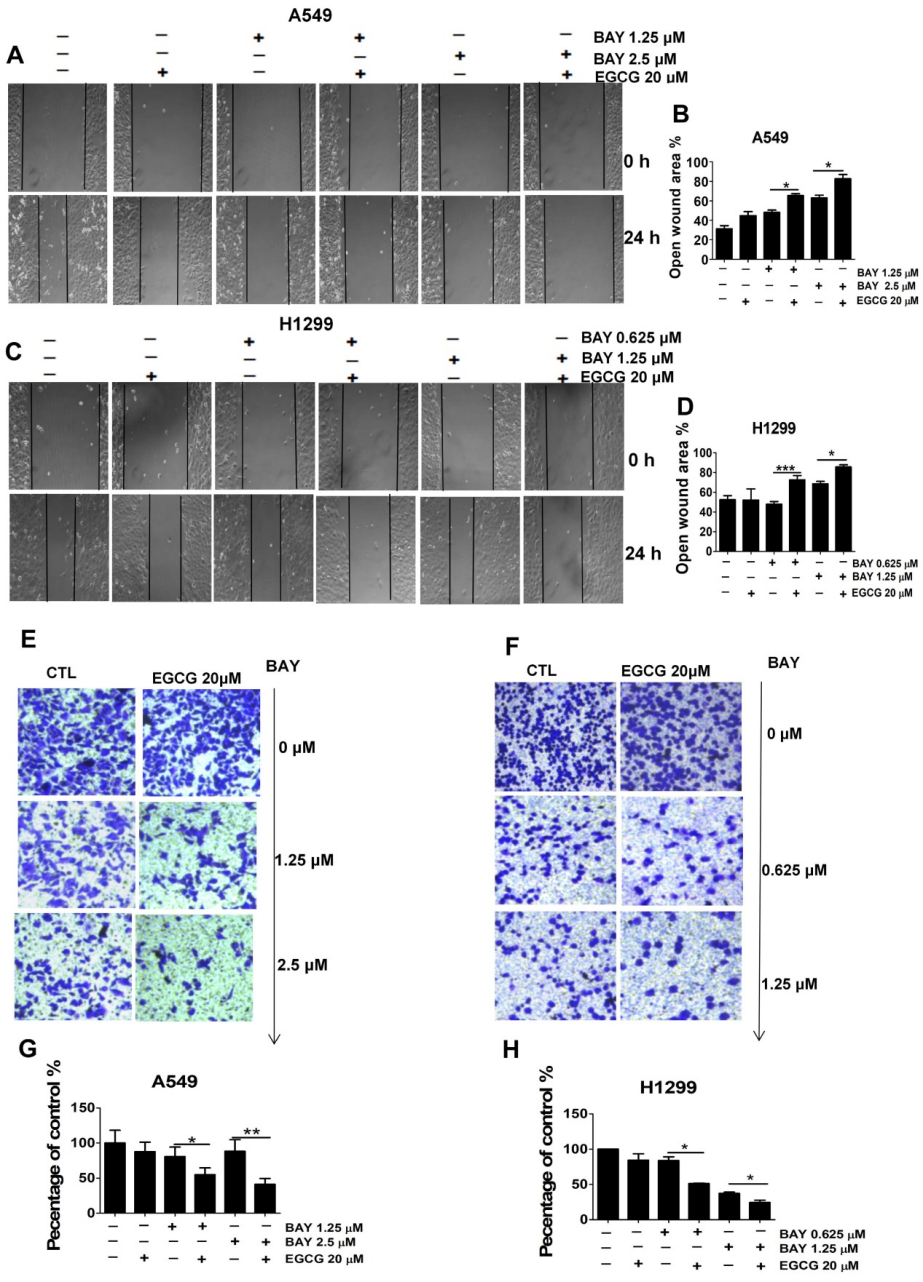
The combination treatment of EGCG and BAY11-7082 inhibits the migration and invasion of lung cancer cells. Representative images of the wounded cell monolayers of A549 (A) and H1299 (C) cells, ×10 for (A, C). (B, D) Quantitative analysis of migration inhibition induced by 24 h treatment with EGCG and BAY-117082. Data are expressed as the percent open wound area compared to untreated cultures. Representative images of the stained A549 (E) and H1299 (F) cells, ×20 for (E-F). (G-H) Quantitative analysis of the anti-invasion activity of EGCG and BAY11-7082. Data are presented as the mean ± S.D. (n=3). * P < 0.05 ** P < 0.01, ***P< 0.001.

NF-κB has been shown to upregulate the expression of matrix metalloproteinases (MMPs), including MMP-2, which is postulated to play a vital role in cancer migration and invasion by the degradation of extracellular matrix (ECM) 32, 33. To determine the effects of EGCG on cancer cell metastasis *in vitro*, we performed wound healing assays and Transwell migration assays. As shown in Fig. 6A-D, EGCG significantly inhibited cell migration in A549 and H1299 cells. When cells were exposed to EGCG (20 μM) co-treatment with BAY11-7082 (0.625 1.25 2.5 μM) for 24 h, the inhibition increased as the concentration increased. In addition, the results of the Transwell migration assays are consistent with the data from the wound healing assays. As shown in Fig. 6E-H, 20 μM EGCG or low concentrations of BAY11-7082 alone did not significantly affect migration in lung cancer cells; however, coadministration of EGCG and BAY11-7082 strongly reduced cell migration. Taken together, these data suggest that cotreatment with EGCG and BAY11-7082 may increase the anti-tumor effects of either drug alone via NF-κB inactivation.

### Coadministration of EGCG and BAY11-7082 has a synergistic antitumor effect in mouse xenograft models

**Figure 7:**
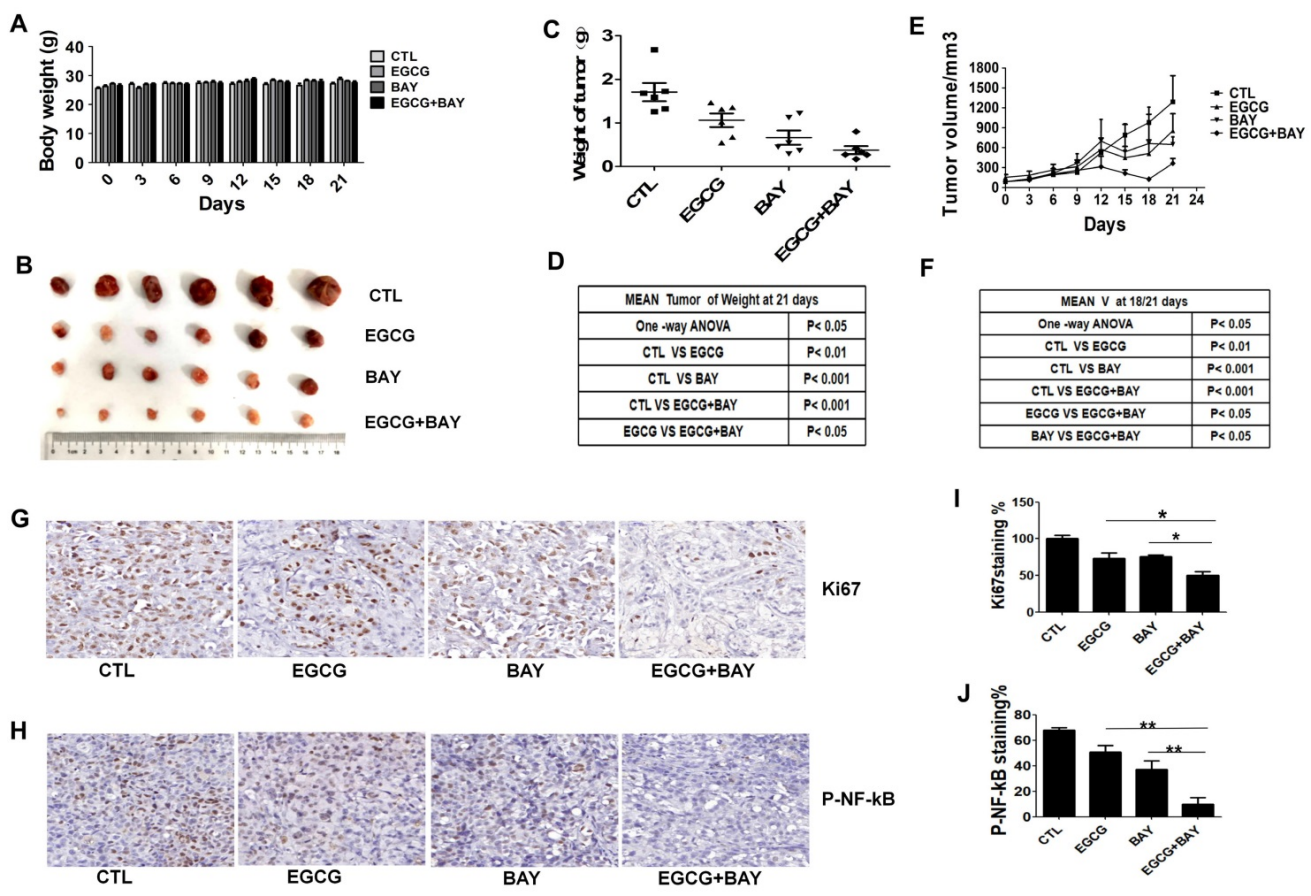
Cotreatment with BAY11-7082 (10mg/kg) enhances the antitumor effects of EGCG (20mg/kg) in an A549 xenograft model. (A) No significant body weight loss was observed during the treatment period in mice. (B) Representative images of tumors from each group at the termination of the experiment. (C-D) Graph showing the tumor weights from the different groups. Cotreatment with EGCG and BAY11-7082 resulted in a significant decrease in tumor weight. (E-F) Graph showing the tumor volumes in each group, which was assessed by caliper measurements and calculated according to the formula length*width*width*0.5. Data are expressed as the mean ± S.E.M., n=6. (G-J) Immunohistochemistry staining (Ki67 and P-NF-kB) of tumor tissues. (G-H) Representative images of IHC staining for Ki67 (G) and P-NF-κB (H) of tumor tissues, ×40 for (G-H). (I-J) Quantitative analysis of the Ki67 (I) and P-NF-κB (J). * P < 0.05 ** P < 0.01.

To determine whether EGCG combined with BAY11-7082 results in significant antitumor effects compared to EGCG alone in* vivo*, we established a mouse xenograft model using A549 cells. After treatment, no significant difference was observed in body weight (Fig. 7A). Tumor growth was suppressed in the EGCG-treated group compared with the control group. However, the group treated with both EGCG and BAY117082 showed a significant decrease in both tumor volume and tumor weight compared with the control group and the EGCG-treated group (Fig. 7B-F). In addition, We performed Ki67 and p-NF-κB assay of xenograft tumor tissues to measure proliferation and NF-κB activity of A549 cells in the xenograft tumor, the results showed in Fig. 7G-J suggested that combination treatment with BAY11-7082 increased the efficacy of EGCG in proliferation (brown color spots number of Ki67 decreased) and NF-κB activity inhibition (brown color spots number of P-NF-κB decreased) to some extent. Comprehensive statistical analysis of these data demonstrated that cotreatment with EGCG and BAY11-7082 significantly improves the antitumor effects of EGCG via inhibiting NF-κB in mouse xenograft models.

## Discussion

Lung cancer is typically treated by surgical removal of the affected tissue followed by radiation therapy and chemotherapy. However, patients often suffer from the serious adverse effects associated with different treatment regimens 4. Throughout the world, tea polyphenol, especially EGCG, has been widely consumed as a health-promoting food ingredient 35. Given its increased popularity and the commercial development of EGCG for cancer treatment 36-39, there is an urgent need to comprehensively study the beneficial effects of EGCG in lung cancer.

In the present study, we found that treatment with EGCG resulted in dose-and time-dependent inhibition of lung cancer cell viability *in vitro*. We next sought to determine whether the antiproliferative effect of EGCG was associated with the induction of apoptosis. Apoptosis plays a crucial role in protecting organisms against tumorigenesis. Many anticancer drugs act to induce apoptosis, eliminating cells that harbor genetic damage or divide inappropriately 40. The results of Western blotting and annexin V-FITC/PI double staining suggested that EGCG induced apoptosis in lung cancer cells in a dose-dependent manner (Fig. 1). Apart from its antiproliferative and apoptosis-inducing effects, EGCG was also found to be effective in inhibiting lung cancer cell migration and invasion at a concentration of 20 μM (noncytotoxic dose) by wound healing and Transwell migration assays (Fig. 6).

To gain insight into the underlying mechanism of EGCG-induced apoptosis several proteins were analyzed, including caspase-3 and -9, cleaved caspase-3 and -9, Bcl-2, NF-κB and p-NF-κB. EGCG effectively activated cleaved caspases-3 and-9 in a dose-dependent manner. EGCG also significantly inhibited the expression of the antiapoptotic protein Bcl-2. In addition, we demonstrated that the expression levels of NF-κB and p-NF-κB were significantly decreased by EGCG in a dose-dependent manner. NF-κB plays important roles in the control of cell growth, differentiation, apoptosis, invasion and angiogenesis by regulating downstream genes, such as Bcl-2, Bcl-xL, survivin, and COX-2 41. Our results clearly show that EGCG inhibits both NF-κB activation and the expression of the downstream target genes Bcl-2, Bcl-xL, and COX-2. These findings correlate well with the observed increase in the apoptotic index. Taken together, these results suggest that EGCG inhibits proliferation and migration and induces apoptosis in A549 and H1299 cells at relatively high concentrations (IC50=86.4 µM for A549 cells and 80.6 µM for H1299 cells), partially via inhibition of the NF-κB signaling pathway.

However, considering the poor absorption and low bioavailability of EGCG, combining this drug with other chemotherapeutic agents may increase its efficacy and utility in lung cancer. Previous reports have indicated that BAY11-7082 can significantly reduce tumor volume and increase survival in mouse lung tumors with high NF-κB activity 26. Thus, our study attempted to further explore whether cotreatment with EGCG and a specific NF-κB inhibitor, BAY11-7082, could enhance its antitumor effects. To our knowledge, this is the first report to analyze the synergistic suppressive effect of EGCG and BAY- 117082 in lung cancer cells.

In a chemoprevention study, the ideal result would be using the lowest concentration of an anti-cancer compound to achieve the best preventive effect. Our preliminary results showed that low concentrations of either EGCG (20 μM) or BAY11-7082 (0.625, 1.25 and 2.5 μM) alone only slightly inhibited tumor growth *in vivo*. However, combined treatment with these two agents markedly suppressed cell growth as detected by MTT and colony formation assays.

Moreover, the observed cell growth inhibition correlated well with apoptosis data, suggesting that cotreatment with EGCG and BAY11-7082 inhibits cell viability partly through the induction of an apoptotic cell death mechanism.

We also showed that combination treatment with EGCG and BAY11-7082 reduced lung cancer cell viability via NF-κB pathway inhibition to a greater degree than treatment with either drug alone. Our data showed that 20 μM EGCG failed to significantly reduce NF-κB phosphorylation. However, in the present study, EGCG was able to reduce the expression of both total and phosphorylated NF-κB. Moreover, immunofluorescence assay of p-NF-κB was performed to observe subcellular location. The result was consistent with the above. In addition, we used luciferase reporter assays to examine NF-κB activity in cells treated with EGCG and BAY11-7082 for 48 h. Our results clearly show that combined treatment with EGCG and BAY11-7082 significantly inhibited NF-κB activity. We also showed that combined treatment with EGCG and BAY11-7082 resulted in downregulation of the downstream target genes Bcl-2, Bcl-XL, MYC, TWIST1, COX2, TNF-a and MMP2, which may have contributed to the inhibition of migration and induction of apoptosis.

In addition to the* in vitro* studies, the antitumor effects of EGCG and BAY11-7082 were investigated in a mouse xenograft model. The combination of EGCG and BAY11-7082 significantly suppressed tumor growth without inducing toxicity (Fig. 7). The results of the* in vivo* study confirmed that cotreatment with EGCG and BAY11-7082 significantly potentiates the antitumor effects of EGCG via down-regulation of NF-κB. Additional studies are required to test the absorption rates of EGCG and BAY11-7082 in animal models and in humans, to determine differences in absorption and bioavailability when administered alone or in combination and to determine the exact mechanisms of these drugs.

## Conclusions

In summary, our results show that EGCG inhibits lung cancer cells by downregulating the expression of NF-κB. We also evaluated the possibility that EGCG combined with BAY11-7082 significantly potentiates this antitumor effect. Considering the poor absorption and low bioavailability of EGCG in clinical trials, we focused on a much lower concentration of this compound. Our results suggested that, even at this low concentration, cotreatment with EGCG and BAY11-7082 could significantly retard tumor progression via down-regulation of NF-κB without any serious side effects. These results collectively suggest that EGCG in combination with BAY11-7082 could be a candidate for the chemoprevention of NSCLC. To our knowledge, this is the first report to detail the enhanced chemopreventive effect caused by EGCG in combination with BAY11-7082 in NSCLC cells.

## Figures and Tables

**Table 1:** Primer sequences of relevant genes used for qRT-PCR.

Gene	Accession #	Sequences of Primers
GAPDH	NM-002046.7	Forward: 5'-AAGGTGAAGGTCGGAGTCAAC-3' Reverse: 5'-GGGGTCATTGATGGCAACAATA-3
BCL2	NM-000657.2	Forward: 5'-GACTTCGCCGAGATGTCCAG-3' Reverse: 5'-GAACTCAAAGAAGGCCACAATC-3'
BCL-XL	NM-138578.3	Forward: 5'-CCCAGAAAGGATACAGCTGG-3' Reverse: 5'-GCGATCCGACTCACCAATAC-3'
COX-2	NM-000963.4	Forward: 5'-GATTGACAGCCCACCAACTT -3' Reverse: 5'-CTCTCCACCGATGACCTGAT-3'
TNFa	NM-000594.4	Forward: 5'-CAGAGGGAAGAGTTCCCCAG-3' Reverse: 5'-CCTTGGTCTGGTAGGAGACG-3'
Cyclin D1	NM-053056.2	Forward: 5'- CTGGCCATGAACTACCTGGA-3' Reverse: 5'-GTCACACTTGATCACTCTGG-3'
C-MYC	NM-00246.7	Forward: 5'-CCAGCAGCGACTCTAAGG-3' Reverse: 5'-CCAAGACGTTGTGTGTTC-3'
TWIST1	NM-000474.4	Forward: 5'-CCATGTCCGCGTCCCACTA-3' Reverse: 5'-CCCACGCCCTGTTTCTTTCTTTGAAT-3'
MMP2	NM_004530.6	Forward: 5'-TGATCTTGACCAGAATACCATCGA -3' Reverse: 5'-GGCTTGCGAGGGAAGAAGTT -3'

## References

[1] Siegel R, Naishadham D, Jemal A (2012). Cancer statistics, 2012. CA Cancer J Clin.

[2] Milligan SA, Burke P, Coleman DT (2009). The Green Tea Polyphenol EGCG Potentiates the Antiproliferative Activity of c-Met and Epidermal Growth Factor Receptor Inhibitors in Non-small Cell Lung Cancer Cells. Clin Cancer Res.

[3] Chen WS, Li Z, Bai L (2012). NF-kappaB, a mediator for lung carcinogenesis and a target for lung cancer prevention and therapy. Front Biosci.

[4] Sunil K D, Manoj K, Devinder KD (2018). Role of EGCG in Containing the ression of Lung Tumorigenesis-A Multistage Targeting Approach. Nutrition and Cancer.

[5] Ke WL, Chen W, Wing YL (2017). EGCG inhibited bladder cancer SW780 cell proliferation and migration both *in vitro* and *in vivo* via down-regulation of NF-κB and MMP-9. J Nutr Biochem.

[6] Naghma K, Hasan M (2010). Cancer and metastasis: prevention and treatment by green tea. Cancer Metastasis Rev.

[7] Chow HH, Cai Y, Hakim IA (2003). Pharmacokinetics and Safety of Green Tea Polyphenols after Multiple-Dose Administration of Epigallocatechin Gallateand Polyphenon E in Healthy Individuals. Clin Cancer Res.

[8] Khan N (2006). Targeting multiple signaling pathways by green tea polyphenol (-)-epigallocatechin-3-gallate. Cancer Res.

[9] Khan N, Afaq F, Saleem M, Ahmad N, Mukhtar H (2006). Targeting multiple signaling pathways by green tea polyphenol (-)-epigallocatechin -3-gallate. Cancer Res.

[10] Aggarwal BB, Shishodia S (2006). Molecular targets of dietary agents for prevention and therapy of cancer. Biochem Pharmacol.

[11] Lin JK, Liang YC (2000). Cancer chemoprevention by tea polyphenols. Proc Natl Sci Counc Repub China B.

[12] Yang GY, Liao J, Li C, Chung J (2000). Effect of black and green tea polyphenols on c-Jun phosphorylation and H2O2 production in transformed and non-transformed human bronchial cell lines: possible mechanisms of cell growth inhibition and apoptosis induction. Carcinogenesis.

[13] Lu G, Liao J, Yang G, Reuhl KR, Hao X, Yang CS (2006). Inhibition of adenoma progression to adenocarcinoma in a 4-(methylnitrosamino)- 1-(3-pyridyl)-1- butanone-induced lung tumorigenesis model in A/J mice by tea polyphenols and caffeine. Cancer Res.

[14] Baud V, Karin M (2009). Is NF-kappaB a good target for cancer therapy? Hopes and pitfalls. Nat Rev Drug Discov.

[15] Karin M, Yamamoto Y, Wang QM (2004). The IKK NF-kappa B system: a treasure trove for drug development. Nat Rev Drug Discov.

[16] Smale ST (2012). Hierarchies of NF-κB target gene regulation. Nat Immunol.

[17] Pires R B, Ferreira G M, Eliana A (2018). NF-kappaB: Two Sides of the Same Coin.

[18] Nakanishi C, Toi M (2005). Nuclear factor-kappaB inhibitors as sensitizers to anticancer drugs. Nat Rev Cancer.

[19] Kan Z, Jaiswal BS, Stinson J, Janakiraman V, Bhatt D (2010). Diverse somatic mutation patterns and pathway alterations in human cancers. Nature.

[20] Mereles D, Hunstein W (2011). Epigallocatechin-3-gallate (EGCG) for Clinical Trials: More Pitfalls than Promises?. Int J Mol Sci.

[21] IKK/NF-κB activation Mol Cells. 2014;37:585-91.

[22] Sadava D, Whitlock E, Kane SE (2007). The greentea polyphenol, epigallocatechin-3-gallate inhibits telomerase and induces apoptosis in drug-resistant lung cancer cells. Biochem Biophys Res Commun.

[23] Tyagi N, De R, Begun J (2017). Cancer therapeutics with epigallocatechin-3-gallate encapsulated in biopolymeric nanoparticles. International Journal of Pharmaceutics.

[24] Saitoh Y, Uota S, Yamamoto N (2010). Overexpression of NF-KB inducing kinase underlies constitutive NF-KB activation in lung cancer cells. Lung Cancer.

[25] Eom DW, Lee JH, Kim YJ (2015). Synergistic effect of curcumin on epigallocatechin gallate-induced anticancer action in PC3 prostate cancer cells. BMB Rep.

[26] Wen X, Etienne M, Oliver TG (2011). Response and resistance to NF-κB inhibitors in mouse models of lung adenocarcinoma. Cancer Discov.

[27] Keller SA, Hernandez-Hopkins D, Vider J, Ponomarev V, Hyjek E, Schattner EJ, Cesarman E (2006). NFkappaB is essential for the progression of KSHV- and EBV-infected lymphomas *in vivo*. Blood.

[28] Li W, Tsen F, Bhatia A (2013). Chapter Five - Extracellular Hsp90 (eHsp90) as the Actual Target in Clinical Trials: Intentionally or Unintentionally. INT REV CEL MOL BIO.

[29] Manohar M, Fatima I, Saxena R, Chandra V, Sankhwar P.L, Dwivedi A (2013). (-)-Epigallocatechin-3-gallate induces apoptosis in human endometrial adenocarcinoma cells via ROS generation and p38 MAP kinase activation. J. Nutr. Biochem.

[30] Li JJ, Gu QH, Li M, Yang HP, Cao LM (2013). Role of Ku70 and Bax in epigallocatechin-3-gallate-induced apoptosis of A549 cells *in vivo*. Oncol Lett.

[31] Sazuka M, Murakami S, Isemura M, Satoh K, Nukiwa T (1995). Inhibitory effects of green tea infusion on *in vitro* invasion and *in vivo* metastasis of mouse lung carcinoma cells. Cancer Lett.

[32] Cheng Z, Lian G, Hao L (2012). Genistein Potentiates the Anti-cancer Effects of Gemcitabine in Human Osteosarcoma via the Downregulation of Akt and Nuclear Factor-kB Pathway. Anti-Cancer Agents in Medicinal Chemistry.

[33] Lee SU, Ahn KS, Sung MH, Park JW, Ryu HW, Lee HJ (2014). Indacaterol inhibits tumor cell invasiveness and MMP-9 expression by suppressing IKK/NF-ĸB activation. Mol Cells.

[34] Hwang BM, Chae HS, Jeong YJ, Lee YR, Noh EM (2013). Protein tyrosine phosphatase controls breast cancer invasion through the expression of matrix metalloproteinase-9. BMB Rep.

[35] Taguchi C, Fukushima Y, Kishimoto Y, Suzuki-Sugihara N, Saita E (2015). Estimated dietary polyphenol intake and major food and beverage sources among elderly Japanese. Nutrients.

[36] Zhang M, Holman CD, Huang JP, Xie X (2007). Green tea and the prevention of breast cancer: a case-control study in Southeast China. Carcinogenesis.

[37] Chhabra SK, Yang CS (2001). Tea and prostate cancer. Epidemiol Rev.

[38] Peschard P, Park M (2007). From Tpr-Met to Met, tumorigenesis and tubes. Oncogene.

[39] Gao YT, McLaughlin JK, Blot WJ, Ji BT, Dai Q (1994). Reduced risk of esophageal cancer associated with green tea consumption. J Natl Cancer Inst.

[40] Fujiki H, Suganuma M, Imai K, Nakachi K (2002). Green tea: cancer preventive beverage and/or drug. Cancer Lett.

[41] Bensassi F, El Golli-Bennour E, Abid-Essefi S (2009). Pathway of deoxynivalenol-induced apoptosis in human colon carcinoma cells. Toxicology.

[42] Vu NT, Park MA, Shultz MD (2016). Caspase-9b Interacts Directly with cIAP1 to Drive Agonist-Independent Activation of NF-kB and Lung Tumorigenesis. Cancer Res.

